# Comprehensive dataset for corporate governance in Oman: Data for a three-level quality assessment of corporate governance

**DOI:** 10.1016/j.dib.2020.106127

**Published:** 2020-08-04

**Authors:** Saeed Rabea Baatwah, Khaled Salmen Aljaaidi, Ehsan Saleh Almoataz

**Affiliations:** a Shaqra University, Saudi Arabia; b Seiyun University, Yemen; c Prince Sattam bin Abdulaziz University, Saudi Arabia; d College of Business Administration, Umm Al-Qura University, Saudi Arabia

**Keywords:** Corporate governance quality, Board of directors, Audit committee, Oman, Dataset

## Abstract

This article describes a dataset on firm-level corporate governance (CG) mechanisms in the Sultanate of Oman. It incorporates, in cross-sectional time series (pooled panel) data, eleven variables: corporate governance quality, board of directors’ quality, audit committee quality, board independence, board expertise, board size, board meetings, audit committee independence, audit committee expertise, audit committee size, and audit committee meetings. The dataset is derived from 1540 firm-year observations for the period 2005 to 2017, excluding financial firms (482) and firms with missing data (29), resulting in a final sample of 1029 firm-year observations. The data were extracted from six sources: OSIRIS database, CG reports, Google, firm's website, directors’ websites, and Bloomberg. Scholarly researchers can readily use this dataset as two data types were created to allow individual and composite measurements of corporate governance mechanisms. The individual determinants are: board independence, board expertise, board size, board meetings, audit committee independence, audit committee expertise, audit committee size, and audit committee meetings. The composite measurements are: corporate governance quality, board of directors’ quality, and audit committee quality. This dataset could be used for investigating corporate governance mechanisms in different business and market issues.

Specifications Table

**Table:** 

Subject area	Accounting, Business, and Management.
Specific subject area	Corporate Governance.
Type of data	Panel Data Table (Excel file).
How data was acquired	We manually collected raw data from six sources: OSIRIS database, CG reports, Google, firm's website, directors’ websites, and Bloomberg.
Data format	Raw, filtered, and combined.
Parameters for data collection	A sample of firms listed on the Muscat Security Market.
Description of data collection	The pooled panel data incorporates at the firm level eleven variables: corporate governance quality, board of directors’ quality, audit committee quality, board independence, board expertise, board size, board meetings, audit committee independence, audit committee expertise, audit committee size, and audit committee meetings.
Data source location	Sultanate of Oman.
Data accessibility	Data are attached with this article.

Value of the Data  

• This dataset panel is valuable for at least two reasons: (1) it includes data on comprehensive corporate governance mechanisms and incorporates the most important main firm-level variables; and (2) the dataset has been organized into two types: (a) individual measurements of board of directors and audit committee characteristics, and (b) composite measures of corporate governance quality, board of directors’ quality, and audit committee quality.
• The data enables academic researchers to examine the individual effect of board of directors and audit committee characteristics on different accounting and auditing issues such as firm performance, financial reporting quality, audit quality, and external corporate governance mechanisms. In addition, it facilitates assessment of the combined effect of board of directors’ quality, audit committee quality, and overall CG quality on these accounting and auditing issues.
• This dataset is also of value to investors, financial analysts, external auditors, bankers, lenders, and capital market regulators in assessing the good corporate governance practices of firms listed on the Muscat Security Market (MSM). This assessment will give them confidence in making different business decisions related to investments, lending, regulation reform and enforcement, and contracting.
• Temporal dynamics and structural breaks may exist because the dataset covers a period of 13 years.
• The data is also useful for studies that have been theoretically framed on agency theory, resource-dependence theory, and/or stewardship theory.

## 1 Data

Using data for an average of 79 firms, this dataset consists of 1029 firm-year observations depicted in cross-sectional time series (pooled panel) data of non-financial firms listed on the MSM in the Sultanate of Oman for the period 2005–2017, as illustrated in Table 1, Panel A. The sampled firms were categorized by the Industry Classifications (2-digit SIC) and year as shown in Table 1, Panel B. These observations represent a wide range of industries, including food and kindred products (SIC code 20), stone, clay, and glass products (SIC code 32), chemical and allied products (SIC code 28), electric, gas, and sanitary services (SIC code 49), and hotels and other lodging places (SIC code 70). For brevity, the supplementary material labeled “Dataset on Governance for Omani Firms” provides the equivalent name for each 2-digit SIC reported in Table 1. Also, the data available in this supplementary material related to governance has been organized in a similar order with the same acronyms as reported in the tables. It is important to note that we used the pooled panel data approach for collecting the data because the number of listed firms on the MSM is small, and there were listed/delisted firms or firms with missing data across the sample period. This approach increases our sampled firms over the balanced or unbalanced panel data approach.^1^ Therefore, discrepancy in the number of firms per year is evident in Table 1.

**Table 1:** Sample distribution for the period 2005–2017.

**Panel A: Sample selection**					
Number of listed firms	1540				
(-) Financial firms	(482)				
(-) Firms with missing data	(29)				
Final sample	1029				

**Panel B: Industry classifications (2-digit SIC) and year for sampled firms**					
**Code**	**No. obs**	**Code**	**No. obs**	**Year**	**No. obs**
01	17	45	2	2005	66
02	39	47	24	2006	81
13	42	48	12	2007	83
20	127	49	98	2008	84
22	27	50	39	2009	83
24	13	51	57	2010	82
26	20	54	13	2011	80
28	77	70	88	2012	79
30	11	73	13	2013	80
32	117	79	2	2014	81
33	39	80	7	2015	81
35	19	82	48	2016	78
36	30	87	24	2017	71
44	24		**1029**		**1029**

Table 2 depicts the descriptive statistics (mean, median, standard deviation, minimum and maximum values, 25 percentiles, and 75 percentiles) for 1029 observations of each corporate governance variable: board independence, board expertise, board size, board meetings, audit committee independence, audit committee expertise, audit committee size, audit committee meetings, corporate governance quality, board of directors’ quality, and audit committee quality. These variables are defined in Table 3.

**Table 2:** Statistics descriptive for the full sample (1029 observations).

Variable	Mean	Median	STD	Min	Max	25 Percentile	75 Percentile
*CGQ*	3.71	4.00	1.65	0.00	7.00	3.00	5.00
*BQ*	1.91	2.00	0.91	0.00	4.00	1.00	3.00
*ACQ*	1.80	2.00	0.98	0.00	4.00	1.00	2.00
*BIND*	0.80	0.86	0.24	0.00	1.00	0.67	1.00
*BEXP*	0.33	0.30	0.27	0.00	1.00	0.13	0.50
*BSZ*	7.05	7.00	1.59	3.00	13.00	6.00	8.00
*BMT*	5.68	5.00	1.95	0.00	17.00	5.00	6.00
*ACIND*	0.88	1.00	0.23	0.00	1.00	0.75	1.00
*ACACEXP*	0.22	0.25	0.23	0.00	1.00	0.00	0.33
*ACSZ*	3.34	3.00	0.68	0.00	6.00	3.00	4.00
*ACMT*	4.77	5.00	1.47	0.00	11.00	4.00	5.00

**Table 3:** Definitions of variables.

Variable	CG quality	Data sources
*CGQ*	= The overall quality of CG which is the aggregation of eight characteristics for board of directors and audit committee.	Developed
*BQ*	= The composite measure for board quality which is the sum of the four board characteristics.	Developed
*ACQ*	= The composite measure for audit committee quality which is the sum of the four audit committee characteristics.	Developed
	**Board characteristics**	
*BIND*	= The proportion of independent directors on the board.	CG report
*BEXP*	= The proportion of directors on the board who are designated as experts. Following [1], we define a director as an expert if he/she has two or more directorships in Omani listed firms.	OSIRIS and CG report
*BSZ*	= The number of directors on the board.	CG report
*BMT*	= The number of meetings held by the board of directors during the year.	CG report
	**Audit Committee (AC) characteristics**	
*ACIND*	= The proportion of independent directors on the AC.	CG report
*ACACEXP*	= The proportion of directors with accounting expertise on the AC. Following [2], accounting expertise is defined as a director who has accounting qualifications, accounting experience (e.g., CFO, accountant), or is an auditor.	OSIRIS, CG report, Google, websites of firms and directors, and Bloomberg
*ACSZ*	= The number of directors on the AC.	CG report
*ACMT*	= The number of meetings held by AC during the year.	CG report

## 2 Experimental design, materials, and methods

We collected data on 11 corporate governance mechanisms manually from the six sources listed above. The operationalization and source of each firm-level corporate governance variable are given in Table 3. The individual determinants are: board independence (*BIND*), board expertise (*BEXP*), board size (*BSZ*), board meetings (*BMT*), audit committee independence (*ACIND*), audit committee expertise (*ACACEXP*), audit committee size (*ACSZ*), and audit committee meetings (*ACMT*). The composite measurements are: corporate governance quality (*CGQ*), board of directors’ quality (*BQ*), and audit committee quality (*ACQ*). The sources, as listed in Table 3, are: OSIRIS database, CG Reports, Google, firm's website, directors’ websites, and Bloomberg.

Basically, the individual characteristics of the board of directors and audit committee are used to build the three measures for CG quality. Thus, we collected raw data for these characteristics based on the original definition commonly used by prior research [e.g., 1; 2; 7]. Data in the supplementary material shows values for these characteristics based on the original definition listed in Table 3. However, we note that these characteristics are mainly measured using a continuous approach. Thus, they should be dichotomized to build the CG quality measures. Following earlier research [e.g., 1; 2], we constructed the quality measures of board quality, audit committee quality, and overall quality of CG after we transferred the continuous measures of *BIND, BEXP, BSZ, BMT, ACIND, ACACEXP, ACSZ,* and *ACMT* to dichotomous variables to reflect high and low quality.^2^ We consider a characteristic equal to or exceeding the splitting point as high quality (assigned one) and others as low quality (assigned zero). This is consistent with the literature that considers boards or audit committees with more independent directors, more experts, larger size, and more diligence as high-quality boards or committees [[1], [2], [3], [4], [5], [6], [7], [8]]. We used splitting points 60% for *BIND* and sample medians for *BEXP, BSZ*, and *BMT* respectively. For *ACIND* and *ACACEXP*, audit committees that are fully independent or have at least one member with accounting expertise are used respectively as cut-off points for these two characteristics. Finally, we used the sample medians as splitting point for *ACSZ* and *ACMT*. We then built the quality measures of CG. For board quality (*BQ*), we summed the dichotomous variables of board of directors’ characteristics whose values range from 0 to 4. As for audit committee quality (*ACQ*), we aggregated the dichotomous variables of audit committee characteristics, again ranging from 0 to 4. Finally, we constructed the overall quality CG (*CGQ*) by summing the eight dichotomous variables of both board of directors’ and audit committee characteristics. The values of *CGQ* can range between 0 and 8.

## Declaration of Competing Interest

The authors declare that they have no known competing financial interests or personal relationships that could have appeared to influence the work reported in this paper.

## Appendix Supplementary materials

Image, application 1

Image, application 2

## Footnotes

1 This type of data will easily allow users to construct balanced or unbalanced panel data. For example, in building balanced panel data, they may choose one year as the starting point and only keep observations for firms from that year over the sampled period. For unbalanced panel data, users simply delete firms with only one observation across the sampled period.

2 We do not include the dichotomous variables for these characteristics in the supplementary material because they are not as crucial as the original data for these characteristics. Further, users may use different points for splitting these characteristics for building the composite or quality measures of CG. Thus, original data for these characteristics gives more flexibility than dichotomous variables.
